# Ultraviolet-B and Heavy Metal-Induced Regulation of Secondary Metabolites in Medicinal Plants: A Review

**DOI:** 10.3390/metabo13030341

**Published:** 2023-02-24

**Authors:** Avantika Pandey, Madhoolika Agrawal, Shashi Bhushan Agrawal

**Affiliations:** Laboratory of Air Pollution and Global Climate Change, Department of Botany, Institute of Science, Banaras Hindu University, Varanasi 221005, India

**Keywords:** gene expression, phenylpropanoids, terpenoids, alkaloids, biosynthesis

## Abstract

Despite a rich history and economic importance, the potential of medicinal plants has not been fully explored under different abiotic stress conditions. Penetration of UV-B radiation and contamination of heavy metals are two important environmental stress for plants with remarkable influence on the defense-related and pharmaceutically important secondary metabolites of medicinal plants. UV-B and heavy metal contamination may become a critical issue that either positively or negatively affects the quality and quantity of secondary metabolites. Such effects may result from changes in the expression level of genes that encode the corresponding enzymes or the inactivation and/or stimulation of specific enzymes involved in the different biosynthetic pathways of the secondary metabolites. Therefore, a comprehensive study of the impact of UV-B and heavy metals individually and in combination on the biosynthesis and accumulation of secondary metabolites in medicinal plants is discussed in the present review.

## 1. Introduction 

Plants are intricate chemical factories that manufacture a myriad of structurally diverse compounds called as ‘secondary metabolites’, which are not vital for the normal development and growth of plants but are crucial for the survival of plants in their various environments [1]. However, the content and composition of these secondary metabolites in plants are impacted by several factors, including environmental variables [1]. These swiftly altering and potentially damaging external environmental variables are constantly interacting with plants [1]. Being immobile organisms, plants have developed sophisticated, convoluted alternative defense mechanisms that use a wide range of secondary metabolites as a tool to combat stressful environments. Among these stresses, heavy metals and UV-B are two important environmental factors. Furthermore, there is strong proof that UV-B radiation facilitates the accumulation of several classes of secondary metabolites such as phenolics, terpenoids, alkaloids and glucosinolates. Many of these UV-B-induced metabolites, if not all of them, are probably involved in UV-B screening and/or reactive oxygen species (ROS) scavenging, which in turn contribute to UV-B protection [2]. Studies have shown that exposure to low doses of UV-B radiation cause stimulation of an enzyme of the secondary metabolites’ biosynthesis pathway (eustress) whereas high doses of UV-B are associated with distress [2]. In general, the balance between the eustress and distress responses of UV-B depends on several factors in addition to UV doses and/or spectral quality, such as exposure time, development stages, plants’ acclimatization strategy, and other environmental variables and genotypes [3,4].

Because of anthropogenic activities, soil’s heavy metal concentration keeps rising as a consequence of growing industrialization, energy production, agricultural operations and the generation of municipal garbage [5]. This has led to increased accumulation of both non-essential metals with unknown physiological and biological functions (Pb, Cd, As, Co, Ag, Cr and Hg) and essential micronutrients (Mo, Cu, Ni, Fe, Mn, Zn and Mg). Essential micronutrients are required for plant growth; however, in excess, they cause perturbation in the growth and metabolism of plants [6]. Toxic levels of heavy metals cause enormous generation of ROS and damage to various biomolecules, leading to perturbation in their growth, physiology and metabolism. Plants are, however, furnished with various defense strategies to overcome stress caused by heavy metals, and chief among them are the secondary metabolites. Kovacik and Klejdus [7] reported that several phenolic metabolites played a pivotal role in the tolerance of medicinal plant *Matricaria chamomilla* against excess of Cd and Cu stress. Oxidative stress imposed by metals also activates a signaling pathway that results in the synthesis of particular secondary metabolites in plants [8,9]. In addition, several studies reported that phytohormones (brassinosteroids, jasmonic acid, salicylic acid, auxin, gibberellins, cytokinin, abscisic acid and ethylene) perform a potential role as signaling molecules and also coordinate different signal transduction pathways in response to abiotic stresses [10]. Choudhary and Agrawal [11] reported that in a leguminous plant *Vigna radiata*, jasmonic acid and salicylic acid increased under UV-B radiation. Nowadays, phytohormones are also utilized exogenously to reduce the negative effect of abiotic stresses or to enhance the secondary metabolites in plants under abiotic stresses. Paeizi et al. [12] reported that methyl jasmonate and Ag^+^ in combination showed the highest content of catharanthine, vindoline, ajmalicine, vincristine and vinblastine in *Catharanthus roseus,* as compared to individual treatments of Ag^+^ and methyl jasmonate. Similarly, the exogenous application of abscisic acid strongly enhanced the accumulation of flavonol in *Vitis vinifera* under UV-B radiation [13]. Consequently, it is crucial to comprehend how medicinal plants respond to UV-B exposure and heavy metal toxicity by adjusting the synthesis of a variety of secondary metabolites. In this regard, the present review focuses on the effect of UV-B and heavy metals on signal transduction and biosynthesis of secondary metabolites, and also on the role of developmental stages of medicinal plants on the accumulation of secondary metabolites under UV-B stress conditions. Furthermore, it also draws attention to the need for studies dealing with the interactive effects of heavy metals and UV-B on the secondary metabolites of medicinal plants. 

## 2. Secondary Metabolites: A Boon from Plants 

Various secondary metabolites are present in plants, and consumers are becoming more interested in them as a result of the extensive usage of plants in the pharmaceutical industry. These secondary metabolites possess various pharmaceutical activities; due to this, they are widely used in the treatment of various disorders of human beings. The WHO estimates that 60% of people worldwide and 80% of human beings in developing countries still rely on herbal remedies. Due to their minimal side effects, the demand for medicinal plants has dramatically increased in recent years, and interest in their use has increased locally, nationally and globally [14]. 

**Figure 1:**
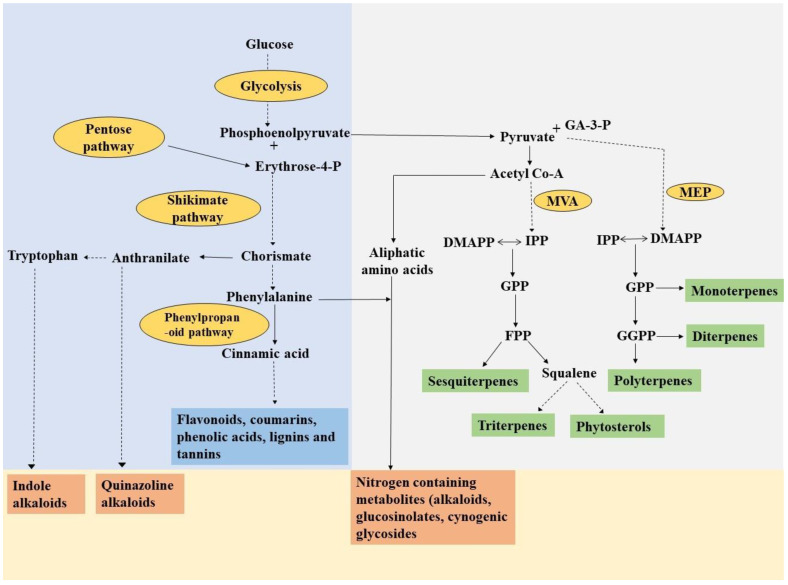
Schematic overview of biosynthesis of different groups of secondary metabolites and their interrelationship with primary metabolism, modified from [14,15]. Abbreviations: MVA- Mevalonic acid pathway; MEP-Methylerythritol phosphate pathway; IPP-Isopentenyl pyrophosphate; DMAPP-Dimethylallyl pyrophosphate; GPP-Geranyl pyrophosphate; FPP-Farnesyl pyrophosphate; GGPP-Geranylgeranyl pyrophosphate.

These secondary metabolites frequently exhibit multiple functionalities and typically contain more than one functional group. Even though secondary metabolites are structurally diverse compounds, they are derived from limited products of primary metabolism [1]. Broadly, secondary metabolites are classified into three groups, phenolics, terpenoids and nitrogen-containing compounds (Figure 1). Terpenoids are synthesized by the plastidic MEP (methylerythritol phosphate) and cytosolic MVA (mevalonic acid) pathway, which utilizes dimethylallyl pyrophosphate (DMAPP) and isopentenyl pyrophosphate (IPP) moieties, respectively, which serve as fundamental building blocks of all isoprenoids. The MEP pathway leads to the generation of mono, di, tetra and polyterpenes, while the MVA pathway results in the synthesis of sesquiterpenes, triterpenes and phytosterols [4,14]. Another important group of secondary metabolites is phenolics which are generally divided into five subgroups, flavonoids, phenolic acid, lignin, coumarins and tannins. Phenolics are synthesized through the phenylpropanoid pathway by utilizing phenylalanine as a key precursor molecule, which in turn is synthesized via the shikimic acid pathway by the reaction of erythrose-4-phosphate and phosphoenolpyruvate (Figure 1). Nitrogen-containing metabolites are also one of the potential groups of secondary metabolites including alkaloids, cyanogenic glycosides and glucosinolates. 

In general, the various essential roles that secondary metabolites play in plant-environment interaction are associated with their occurrence in a variety of cellular and subcellular compartments. Agati et al. [16] revealed that dihydroxy B-ring-substituted flavonoids localized to the chloroplasts, vacuoles and the nucleus, and may effectively scavenge ROS from these sites. In addition, Facchini [17] reported that several enzymes that are involved in the biosynthesis pathway of different alkaloids localized in various subcellular compartments, such as enzymes of vindoline (a terpenoid indole alkaloid) biosynthesis in *Catharanthus roseus,* are localized to not less than five subcellular compartments. Similarly, the biosynthesis of quinolizidine alkaloids in a legume, namely lupin, occurs in the chloroplast of mesophyll cells [17]. Much less numerous are the studies related to localization of secondary metabolites in subcellular compartments concerning the effects of abiotic stresses, particularly UV-B and heavy metals. Zagoskina et al. [18] observed that, exposure of the callus culture of *Camellia sinensis* to UV-B causes enhanced accumulation of phenolic acids in intercellular spaces and cell walls, and accumulation of lignin on the surface of the callus culture [18]. 

## 3. UV-B Perception, Signal Transduction and Regulation of Genes of Secondary Metabolites’ Biosynthesis Pathway

**Figure 2:**
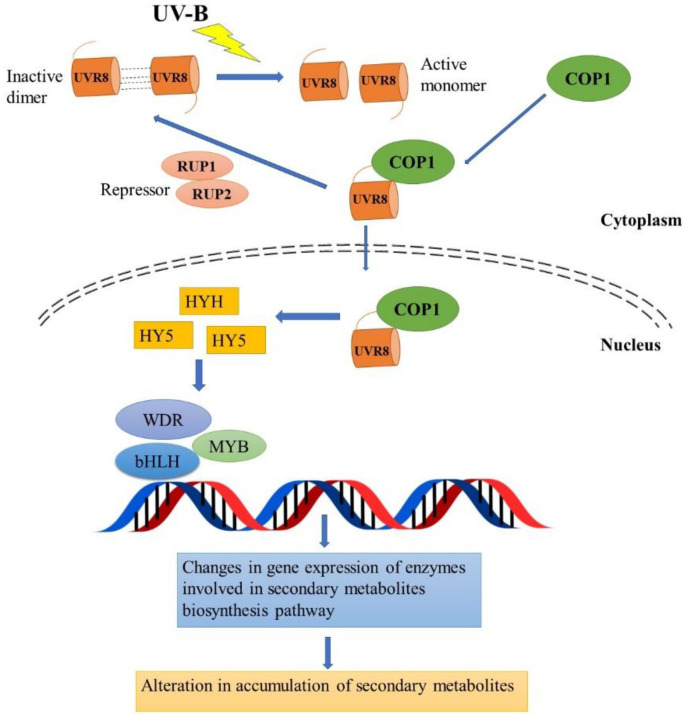
UVR8-mediated signal transduction pathway of secondary metabolites’ biosynthesis in plants, modified from [20,22] UV-B-ultraviolet-B radiation; UVR8-A UV-B receptor; COP1-constitutively photomorphogenic 1; HY5-elongated hypocotyl 5; HYH-HY5 Homolog; MYB transcription factor-V-MYB myeloblastosis viral oncogene homolog; bHLH-Basic helix loop–helix; WDR, Tryptophan–aspartic acid repeat; RUP1/2,-repressor of ultraviolet-B photomorphogenesis.

UV-B radiation is one of the potential environmental factors that can regulate the production of secondary metabolites through induction/inhibition of the corresponding genes of the biosynthetic pathway at the transcriptional level [19]. It is suggested that, following the perception of signals, transcription factors bind to the relevant cis-elements located in the promoter regions of the genes they are intended to activate/repress, or they combine with other transcription factors to set up a regulatory complex. These transcription factors tightly control both expressions of transcripts from related biosynthetic genes and the inducible production of secondary metabolites [19]. Plants perceive UV-B radiation via a specific UV-B receptor, namely UVR8 and various other elements that are involved in the downstream signaling cascade [20]. The majority of research on Arabidopsis revealed that the UVR8 photoreceptor is a 440 amino acid residue with a seven-bladed β-propeller core, a flexible 60 amino acid c-terminal region and a short N-terminal extension [21]. In the cytosol, the UVR8 photoreceptor occurs as a homodimer; however, when exposed to UV-B radiation, it rapidly undergoes monomerization mediated by the tryptophan molecules that serve as the UV-B chromophore. A multifunctional E3 ubiquitin ligase called COP1 (constitutive photomorphogenic 1) further binds with the activated UVR8 monomer. Additionally, repressor of UV-B photomorphogenesis 1 (RUP1) and RUP2 (WD40-repeat proteins), inactivate this pathway by disturbing UVR8-COP1 interaction, and again re-dimerize UVR8 monomers through a negative feedback loop to regenerate UVR8 homodimer (Figure 2). 

COP1 performs a crucial role in the accumulation of UVR8 in the nucleus, and acts as a positive regulator of UV-B signaling by transducing the changes in expression and function of several genes or transcription factors that participate in the regulation of metabolic and physiological processes [19]. COP1 alone inhibits photomorphogenesis in plants by encouraging the degradation of a transcription factor HY5 (elongated hypocotyl 5). However, when COP1 interacts with the UVR8 monomer under UV-B radiation, HY5 is shielded from COP1-mediated degradation. Hence, the complex of UVR8-COP1 stabilizes HY5 and consecutively HY5 and HYH (HY5 homolog) induce the activity of various other transcription factors for the transcription of genes necessary in the biosynthesis of secondary metabolites (Figure 2). Among these transcription factors, the WDR, bHLH and MYB family are known to regulate the various secondary metabolite biosynthesis pathways including flavonoid biosynthesis in several plant species. It was found that in Arabidopsis, HY5 directly activates a group of R2R3-MYB transcription factor-encoding genes, such as *MYB11*, *MYB12* and *MYB111,* and these are responsible for the expression of several flavonoid biosynthetic genes including *CHI*, *CHS* and *FLS1* under UV-B radiation [23]. Yang et al. [24] revealed that CmUVR8, COP1 and HY5 played an essential role in UV-B mediated expression of the flavonoid’s biosynthesis pathway genes, leading to enhanced accumulation of several flavonoids in the medicinally important plant *Chrysanthemum morifolium*. Many of these studies have shown that following exposure to UV-B radiation, UVR8 is upregulated and further influences the signal transduction pathway downstream of UV-B perception. In contrast, Shamala et al. [25] revealed that in *Camellia sinensis*, UVR8 was downregulated and the transcript level of HY5, bHLH62, MYB4 and MYB12 transcription factors (that regulate the downstream structural genes) were differentially affected under both short- and long-term exposure to UV-B radiation.

## 4. UV-B Mediated Biosynthesis and Accumulation of Secondary Metabolites in Medicinal Plants

UV-B-mediated alteration in the secondary metabolites from different biosynthetic pathways (phenylpropanoids, terpenoids and alkaloids) has been extensively studied in various plant species including medicinal plants (Table 1). 

However, the alteration in the metabolome of plants under UV-B radiation is a sophisticated stress-induced modification that is largely influenced by species, genotypes, th developmental stage of plants, the dynamics of UV-B exposure and various other environmental factors [19]. Furthermore, exposure of plants to UV-B radiation is also correlated with the pace of CO_2_ assimilation by which carbon is diverted from the primary metabolism to the secondary metabolism [43]. In order to achieve this, UV-B-induced enzymes such as phenylalanine ammonium lyase (PAL) and other enzymes that sequentially convert the precursor of phenylpropanoids (i.e., phenylalanine) to various other phenolics played a pivotal role in shifting carbon for secondary metabolites’ biosynthesis. Liu et al. [44] reported that UV-B radiation induced a shift from carbon assimilation to carbon accumulation and involved the reprogramming of metabolism to shift carbon towards the phenylpropanoid biosynthesis pathway in *Astragalus mongholicus*. A study carried out by Zhang et al. [32] on *Glycyrrhiza uralensis* showed that the content of phenylalanine and the expression level of unigenes encoding PAL and C4H were upregulated under UV-B radiation, which indicated that UV-B promotes the conversion of phenylalanine into flavonoids by enhancing the expression level of *PAL* and *C4H* under UV-B radiation in *G. uralensis*. 

In *Pelarogonium zonale*, which is a model plant for investigating interactions between sources and sinks within the same leaf, UV-B radiation at a dose of 0.90 W m^–2^ leads to differential responses in variegated green and white leaves. In order to compensate for the loss of photosynthetic activity and production of flavonoids in white tissue, UV-B light stimulates the allocation of C from source to sink, i.e., from green tissues to white tissues [45]. Moreover, UV-B radiation of 4 W m^−2^ and 6 W m^−2^ with continuous and 16 h irradiation on *Perilla frutescens* showed enhanced expression of *PAL*, *RAS* and *TAT* genes related to the rosmarinic acid biosynthetic pathway after long exposure to UV-B, and was correlated with an enhanced content of rosmarinic acid. However, the expression of *CHS*, *ANS*, *F3H* and *DFR* genes related to the biosynthesis of anthocyanin decreased under 16–48 h UV-B irradiation, which lead to a reduction in anthocyanins content in *P*. *frutescens* [34]. UV-B radiation at two doses: 1.9 (medium dose) and 3.4 kJ m^−2^ d^−1^ (high dose) enhanced the content of saponin namely eleutheroside B and E by 1.5 and 1.9 times, respectively under medium dose, and by 1.9 and 3.5 times as compared to control at a higher dose in *Acanthopanax senticosus* (which is commonly used as anti-diabetic, neuroprotective, anti-inflammatory, cardioprotective agent). This implies that the therapeutic properties of *A. senticosus* can be enhanced to some extent by UV-B radiation [35]. *Artimisia annua* is a medicinal plant of the family Asteraceae and possesses active constituent artemisinin (a sesquiterpene lactone), other sesquiterpenes and flavonoids. It is effective against *Plasmodium falciparum* and other malaria-causing parasites, including those resistant to chloroquine and quinine. 

In a study by Pandey et al. [37] on *A. annua*, UV-B radiation at a dose of 2.8 W m^−2^ upregulates the transcript of artemisinin biosynthesis pathway genes such as *HMGR*, *IPPi*, *DXR*, *ADS*, *CYP71AV1*, *FPS* and *RED1* genes, leading to greater accumulation of artemisinin. However, the expression of genes such as *ECS*, *BFS* and *QHS* encoding 8-epicedrol synthase, β-farnesene synthase and β-caryophyllene synthase, which is involved in the production of other sesquiterpenes (8-epicedrol, β-farnesene and β-caryophyllene), apart from artemisinin, was down-regulated. Downregulation of *ECS*, *BFS* and *QHS* transcripts suggested that FPP (precursor of all sesquiterpenes) was utilized for the biosynthesis of artemisinin in *A. annua* under UV-B radiation. Another study carried out by Pan and coworkers [46] at a lower dose (1.44 kJ m^−2^ d^−1^) of UV-B on *A. annua* also reported the upregulated expression of enzymes such as *CYP71AV1*, *ADS* and the related WRKY transcription factors that are responsible for enhanced accumulation of artemisinin under low dose of UV-B [46]. *Achyranthes bidentata* is utilized as an anti-inflammatory, diuretic, antibacterial and anti-arthritic agent due to the presence of active metabolites oleanolic acid and ecdysterone. The biosynthesis of ecdysterone and oleanolic acid occurs through the MVA pathway through the conversion of isopentenyl pyrophosphate to 2,3-oxidosqualene. Li et al. [40] reported enhanced expression of *HMGR*, *HMGS*, *PMK*, *SE*, *SS*, *FPS*, *β-AS* and *CAS* genes of the oleanolic acid and ecdysterone biosynthesis pathway that ultimately leads to enhanced accumulation of ecdysterone and oleanolic acid in *A. bidentata* under UV-B radiation. Furthermore, it was reported that UV-B mediated accumulation of secondary metabolites showed variable responses depending on cultivars. Inostroza-Blancheteau et al. [36] reported that UV-B radiation differentially affects the gene expression of PAL, CHS, ANS and F3′H enzymes involved in the phenylpropanoid pathway in two cultivars (Brigitta and Bluegold) of *Vaccinum corymbosum*. As previously mentioned, experimental conditions also affect the UV-B-mediated accumulation of secondary metabolites in plants. Dolzhenko et al. [47] performed an experiment in field and growth chamber conditions to observe whether UV-B differentially affects the flavonoids and terpenoids metabolism in *Mentha × piperita* in two conditions. Under UV-B radiation, there is an interplay between flavonoids and terpenoids depending on growth conditions, as revealed by a higher content of terpenoids but a lower content of total phenol in *M. piperita* grown under a growth chamber. However, a reduced amount of terpenoids with a concomitant increase of phenol content in field-grown plants was observed. 

Although there are several studies that have revealed the positive or stimulatory effect of UV-B on secondary metabolites of medicinal plants, some studies also documented the inhibitory or non-significant effect of UV-B. *Sinopodophyllum hexandrum*, is a medicinally important plant and widely found in the mountain area of the Himalayan region. Its rhizome possesses an active constituent podophyllotoxin that is utilized as an anticancer agent. Lv et al. [38] reported that under UV-B, the expression of genes of sugar (*β-1,3-glucanase* and *XET*) and flavonoid biosynthesis (*PAL*, *DTX41* and *CHS1*) were enhanced, and this was responsible for enhanced accumulation of sugar and flavonoids. However, the expression level of genes related to the biosynthesis of podophyllotoxin (*C3H*, *OMT3* and *2-ODD*) decreased, consequently leading to a reduced content of podophyllotoxin under UV-B. So, it might be suggested that *S. hexandrum* tried to adapt under UV-B at high altitudes by allocating its resources towards the development of the root and UV-B screening metabolites (flavonoids) at the expense of its active constituent, i.e., podophyllotoxin. Furthermore, the seeds of a tropical shrub species, *Bixa orellana* accumulate a high amount of a secondary metabolite called bixin, which is an apocarotenoid and formed by the oxidative cleavage of carotenoids. Due to the presence of bixin, *B. orellana* possesses medicinal properties such as apoptotic, anticancer, antitumor and antioxidant activity. The mRNA expression of the genes that participated in bixin biosynthesis pathway namely *PSY*, *DXS*, *PDS*, β-*LCY* and ε-*LCY* showed differential expression under UV-B radiation. The total carotenoid content decreased; however, no significant changes occurred in the content of bixin under UV-B-treated seedlings of *B. orellana* [48]. A study carried out by Rodriguez-Morrison et al. [49] on *Cannabis sativa* under different doses of biologically effective UV-B radiation (0.16–13 kJ m^−2^ d^−1^) also reported a reduction in the content of active metabolites such as tetrahydrocannabinol and cannabidiol. Choi et al. [41] observed non-significant variation in the active metabolites, ginsenosides (a saponin used as an anticancer, immunomodulatory and anti-stressor agent) of *Panax ginseng* under different doses (0.36, 0.72 and 1.08 kJ m^−2^ d^−1^) of UV-B. 

## 5. Role of Developmental Stages on Accumulation of Secondary Metabolites in Medicinal Plants under UV-B Stress

Due to the evident variations in the life cycles of plants, a significant number of secondary metabolites frequently occur at a certain stage of plant development. Several studies have reported that the accumulation of secondary metabolites under UV-B stress conditions are largely influenced by the developmental stages of medicinal plants. Sun et al. [50] reported that UV-B duration and the appropriate stage of leaf development were two important key factors that influence the secondary metabolites of *Ginkgo biloba*. Maximum accumulation of flavonoids was found to occur in the younger leaves of *G. biloba* under moderate exposure to UV-B radiation [45]. A study devised by Rai and Agrawal [51] under the intermittent and continuous treatment of elevated UV-B (ambient ± 7.2 kJ m^−2^ d^−1^) on three developmental stages (45, 75 and 110 DAG; days after germination) of *Eclipta alba* reported a maximum content of wedelolactone in leaves at 75 DAG under intermittent treatment. However, at a later developmental stage (110 DAG), the wedelolactone content showed a reduction under both treatments; this might have occurred due to the degradation of its precursor over an extended period of time. Rizzi et al. [52] also stated that the content of phenolic acids, flavonoids and total phenols was higher in young leaves of *Salvia verticillata* exposed to UV-B radiation. 

Contrary to this, in a perennial herb *Adhatoda vasica*, the content of two important quinazoline alkaloids, namely vasicoline and vasicinone, increases with an increase in the age of the plant. Further, the content of both alkaloids was maximum under the treatment of UV-B at the reproductive stage of *A. vasica* [4]. Moreover, the content of different metabolites of a plant species could not be always the same at different developmental stages as in *Prunella vulgaris*. It contains several important metabolites, including rosmarinic acid, caffeic acid, flavonoids, hyperoside and salviaflaside. The content of these metabolites such as caffeic acid, rosmarinic acid, hyperoside and flavonoids was higher at different developmental stages and reduced during its development, i.e., from bud to mature fruiting stage, while salviaflaside showed enhancement during development. In addition, UV-B significantly increased the content of secondary metabolites and antioxidant activity of *P. vulgaris* at all developmental stages [53]. Similarly, younger leaves of *Centella asiatica* have higher levels of saponins, whereas older leaves have higher levels of sapogenins. However, UV-B radiation causes non-significant changes in the content of both sapogenin and saponin in *C. asiatica* [54]. 

## 6. Impact of Heavy Metals on Signaling, Biosynthesis and Accumulation of Secondary Metabolites in Medicinal Plants 

Contamination of heavy metals in the environment due to diverse human activities is a serious environmental issue. The biosynthesis of secondary metabolites may eventually be impacted by the cultivation of medicinal plants in heavy metal-contaminated environments, leading to dramatic alterations in the quantity and quality of these substances [55]. Under heavy metal stress, there is enhanced production of reactive oxygen species (ROS) that can lead to peroxidation of the lipid membrane, damage to macromolecules and oxidative stress. However, besides their damaging activity, ROS also act as signaling molecules that can activate further downstream signaling pathways of secondary metabolite biosynthesis in plants. It was found that enhanced ROS production as a result of heavy metal stress activates the mitogen-activated protein kinases (MAPKs). Further, MAPKs modulate the expression of genes that participated in secondary metabolism and defensive responses via direct phosphorylation of the downstream transcription factors. Paeizi et al. [12] demonstrated that the application of Ag^+^ to *Catharanthus roseus* caused enhanced ROS production, which further triggered the activation of CrMAPK3. In addition, overexpression of CrMAPK3 leads to upregulation of ORAC3 (Octadecanoid responsive *Catharanthus* AP2-domain3), which is a key regulator transcription factor of the genes of the terpenoid indole alkaloids’ (TIA) biosynthesis pathway in *C. roseus*. Upregulation of transcription factors (CrMAPK3 and ORAC3) under Ag^+^ treatment was correlated with enhanced expression of the genes of the TIA biosynthesis pathway such as *STR*, *DAT*, *GS* and *CrPRX1* that ultimately leads to an increment in the content of TIAs such as vincristine, catharanthine, indoline, ajmalicine and vinblastine in *C. roseus*. 

**Figure 3:**
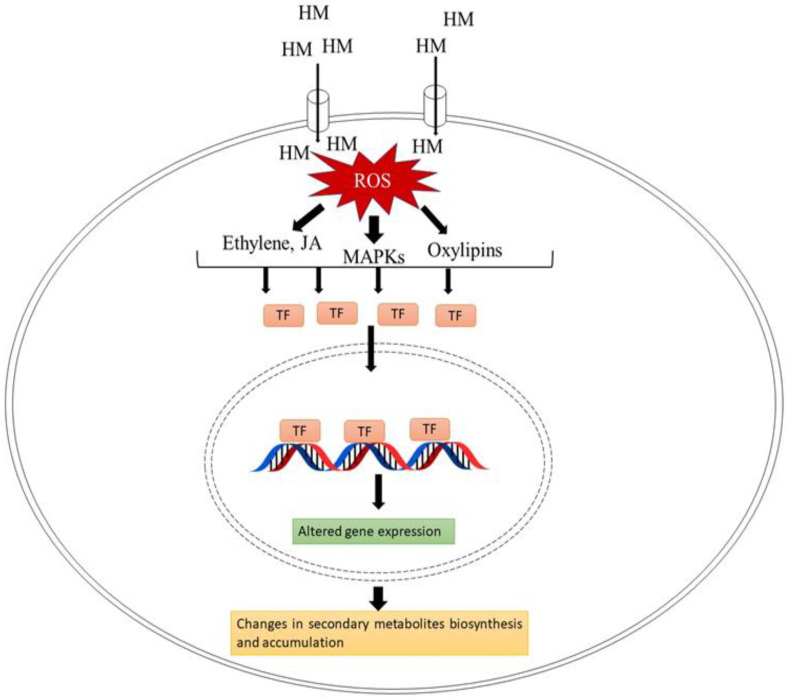
Diagrammatic representation of heavy metal-induced signal transduction in plants, adapted from [8,57]. Abbreviations: HM-Heavy metals; ROS-reactive oxygen species; MAPK-mitogen activated protein kinase; TF-transcription factors.

In addition, enhanced ROS production causes peroxidation of polyunsaturated fatty acids (PUFA) of membranes, which eventually leads to the production of extremely active signaling molecules. Fatty hydroperoxide production results in the production of oxylipins (oxygenated fatty acids), which is a group of active signaling molecules that support plant defense responses and activate genes taking part in the synthesis and accumulation of secondary metabolites [12,56]. Heavy metals are also very well known to activate the expression of other signaling molecules. It was found that in Ag^+^-exposed hairy root cultures of *Brugmansia candida* ethylene play a role in the regulation of pathways that results in the formation of tropane alkaloids (hyoscyamine and scopolamine) [56]. Figure 3 shows a diagrammatic illustration of the signaling pathway involved in heavy metal-induced secondary metabolite accumulation. 

Nowadays, heavy metals may also be utilized as elicitors to enhance the content of secondary metabolites in medicinal plants. Among these, Ag^+^ is regarded as an efficient elicitor to induce bioactive compounds in medicinal plants. Xing et al. [58] reported that Ag^+^ (15 μM) effectively enhances the accumulation of two major bioactive constituents, phenolic acids and tanshinones, in *Salvia miltiorrhiza* hairy roots via upregulation of the genes of enzymes that participated in their biosynthesis pathway. Another study by Pesaraklu et al. [59] at the same dose of Ag^+^ (15 μM) on two different species of *Salvia*, i.e., *S*. *officinalis* and *S*. *verticillata*, also reported enhancement in the total phenolic acid content by 1.60 and 1.52-fold, respectively, as compared to control. Increasing the concentration of Cd in soil increased the activity of phenylpropanoid pathway enzymes such as PAL and CAD by 1.82 and 6.48-fold as compared to control at 100 and 300 µM Cd concentration; this consequently led to higher accumulation of flavonoids and total phenolics in *Withania somnifera* [60]. Rai et al. [61] carried out an experiment in hydroponics and soil conditions to observe the effect of As on the production of artemisinin, and reported that As stress stimulated the content of artemisinin in *Artimisia annua* in a dose-dependent manner. According to their findings, As stress raised the artemisinin content in *A. annua* in either of two ways: (i) through metabolic adjustment by involving conversion of dihydroartemisinic acid (the immediate precursor of artemisinin) to artemisinin as a consequence of enhanced ROS production, or (ii) by overexpression of genes of enzymes involved in artemisinin biosynthesis, such as *ADS*, *HMGR*, *FDS* and *CPYA171*. Rai et al. [61] further suggested that *A. annua* may be considered a promising option for planting in arsenic-polluted areas for enhanced artemisinin production. 

*Centella asiatica* is frequently used for memory loss, nootropic disorders and insomnia, and is rich in triterpene glycosides called centellosides (madecassoside, asiaticoside and asiatic acid). Biswas et al. [62] performed an experiment in soil by applying different doses of Cd nitrate (50, 100, 150 and 200 mg Kg^−1^) and revealed that *C. asiatica* is quite tolerant to applied doses of Cd, as it showed enhanced biomass and significant accumulation of Cd in roots and shoots at the higher dose. Furthermore, the transcript of three biosynthetic genes that are involved in the production of centellosides such as *BAS*, *SQS* and *CAS* showed maximum upregulation at higher doses, leading to a higher accumulation of centellosides. Based on the study, Biswas et al. [62] suggested that *C. asiatica* can be utilized as a phytostabilizer (as it showed higher accumulation of Cd in roots and lowered translocation in the shoots) with additional pharmaceutical benefits and without rigorous post-harvest processing to remove Cd. 

In a study carried out by Ibrahim et al. [63] under the individual and combined effect of Cu and Cd, it was observed that both heavy metals individually enhance the content of phenolics, flavonoids and saponins; hence, the medicinal property of *Gynura procumbens*, as demonstrated by higher antibacterial activity under Cd and Cu stress. In contrast, a combination of Cd and Cu caused a reduction in these metabolites, leading to reduced antibacterial activity. Besides an increase in the secondary metabolites and medicinal properties of *G. procumbens* under individual treatment of Cd and Cu, Ibrahim et al. [63] reported that it was not advisable to consume *G. procumbens* in areas with contamination of Cu and Cd due to their accumulation above the safety limit recommended by the WHO (i.e., 1.3 and 0.3 mg kg^−1^, respectively). 

Furthermore, some studies also reported the negative effect of heavy metals on secondary metabolites in medicinal plants. *Panax notoginseng* saponins (PNS) are important secondary metabolites of *Panax notoginseng* which is synthesized through the terpenoid biosynthesis pathway. It was found that Cd stress reduced the accumulation of these saponins in *P. notoginseng* as a result of the downregulated expression of *PMK*, *MVK* and *GGPS* of the terpenoid backbone biosynthesis pathway [64]. *Hypericum perforatum* contains three important secondary metabolites, hypericin, hyperforin and pseudohypericin, due to which it is widely utilized in the treatment of neurological disorders, cancer and tumors. *H. perforatum* grown under nickel supplements of 25 or 50 mM, showed a 15–20-fold reduction in the quantity of hypericin and pseudohypericin. However, *H. perforatum* entirely lost its ability to synthesize or store hyperforin under Ni stress, suggesting that Ni toxicity can alter its chemical makeup and have a significant negative impact on the quality, safety and effectiveness of natural products made by this plant species [65]. Another study carried out by Babula et al. [66] on *H. perforatum* under Cd and La stress (10 µM) also revealed a significant reduction in hypericin content under La and Cd + La stress which was further correlated with a reduction in the expression of its putative *hyp-1* gene. However, the content of hyperforin increased significantly under both Cd and La stress as compared to the control. In addition, *Ligusticum chuanxiong,* which is used widely in traditional Chinese medicine for curing neurological and respiratory disorders, also showed a reduction in its active metabolites, tetramethylpyrazine, ferulic acid and ligustilide, under single and combined exposure to Cd and Pb. However, this reduction was greater under individual treatments as compared to their combined treatments [67]. 

Some aromatic and medicinal plants that are rich in essential oils can absorb and accumulate metal pollutants in the harvestable part, making them a viable option for polluted site rehabilitation without contaminating essential oils [55]. Heavy metals are not eliminated from the plant tissues during essential oil extraction by distillation, and instead remain in the extracted residues of the plant, thereby limiting the detectable amounts of heavy metals in the essential oil. Thus, in contrast to other edible crops and medicinal plants, planting these aromatic plants in areas with metal contamination may not cause heavy metals to enter the food chain, and may not have a negative economic impact [55]. 

A pot study was devised by Ahmad et al. [68] to observe the performance of the pharmaceutically important essential oil-producing plants *Mentha piperita* (peppermint) at different doses of Cd (30–120 mg kg^−1^ soil). All the applied doses of Cd enhanced the concentration of essential oil with a maximum enhancement of 16.1% at 60 mg kg^−1^ Cd. The active constituent menthol was found to be enhanced at lower doses (30 and 60 mg kg^−1^), whereas it showed a 3% reduction at the higher dose (120 mg kg^−1^) of Cd. In addition, Prasad et al. [69] observed the effect of two levels of Cr (30 and 60 mg kg^−1^ soil) on the composition and yield of essential oil of three mint species (*Mentha piperita*, *M. arvensis* and *M. citrata*). The authors reported that the content of essential oil significantly declined in all mint species with Cr application, except *M. citrata*, in which the essential oil content did not significantly vary with 30 mg kg^−1^ Cr. Menthol was found to be non-significantly varied with both levels of Cr. Given that heavy metals may likely serve as a stimulus for the production of secondary metabolites in medicinal plants, growing these plants in soils contaminated with heavy metals may be advantageous, if the plants are capable of withstanding high levels of soil heavy metal, for secondary metabolite production [62]. Table 2 compiles the studies on the accumulation of secondary metabolites in different medicinal plants under heavy metal stress. 

## 7. Interactive Effect of UV-B and Heavy Metals on Secondary Metabolites of Medicinal Plants 

In the environment, plants are generally subjected to various stresses, and the effect of one stress may be intensified or alleviated by simultaneous action of another stress. Following this, Gondor et al. [73] reported that UV-B radiation may either have an adverse or beneficial influence on wheat based on other abiotic stresses such as Cd or drought. Furthermore, it was also reported by Reiger et al. [74] that increased UV-B radiation causes co-tolerance to Hg stress at the physiological and transcriptional level in *Elodea nuttallii* subjected to UV-B and Hg stress. Several studies reported that the alteration in the membrane permeability of UV-B exposed plants may facilitate the enhanced uptake of heavy metals, leading to a more pronounced effect on plants with a combination of two stresses.

Besides the interactive effect of heavy metals and UV-B on plants, there are not many data available regarding their interactive effect on the secondary metabolites of medicinally important plants. Pandey et al. [75] reported the maximum content of psoralen (a furanocoumarin, used in the treatment of psoriasis, vitiligo and leukoderma) in seeds of *Psoralea corylifolia* under combined treatment of Cr and UV-B. Further, Pandey et al. [75] suggested that *P. corylifolia* can be grown in an area with a high flux of UV-B and Cr contamination due to the higher content of psoralen and accumulation of Cr below the permissible limit in *P. corylifolia* seeds. Rojas-Lillo et al. [76] also revealed that the anthocyanin and phenol content of *Vaccinium corymbosum* were maximum under the combined treatment of Mn and UV-B. Vasicine (a pyrroquinazoline class of alkaloids) is a major active metabolite of *Adhatoda vasica* which is used to treat tuberculosis, cough and respiratory diseases. Under individual and combined treatment with UV-B and Cr, the content of vasicine increased, with maximum enhancement under a combined treatment of UV-B and Cr [77]. In addition, a study carried out by Azadi et al. [78] on *Thymus vulgaris* involving silver nanoparticles (50 and 100 mg L^−1^ AgNPs) and UV-B exposure (30 and 60 min) reported that, AgNPs alleviate the negative effect of UV-B on the plant’s yield of essential oil and composition to some extent. The plant’s essential oil yield was maximum under a combined treatment of a lower dose of UV-B (i.e., UV-30) and a higher dose of AgNPs (100 mg L^−1^). The major active metabolite of essential oil, thymol, was found to be enhanced maximally under higher doses of both UV-B and AgNPs (UV60NA100). As the exact mechanism underlying the interaction between UV-B and heavy metals on secondary metabolites is not clear, it might be possible that to cope with both stresses, plants divert more of their photosynthates to the production of secondary metabolites. It is also possible that there is cross-talk in the signaling pathways of these two abiotic stresses, which might be responsible for higher expression of the genes involved in the biosynthesis pathway, leading to enhanced production of secondary metabolites.

## 8. Conclusions and Future Prospects

The studies reviewed in this article showed that UV-B and heavy metals cause alterations in the secondary metabolites of medicinal plants. Furthermore, it was found that UV-B and heavy metal stress activate a signal transduction pathway that consequently leads to alterations in the expression of various genes of the enzymes taking part in the biosynthesis of secondary metabolites in medicinal plants. However, these alterations in biosynthesis and accumulation of secondary metabolites range from having positive to negative to no effects, depending on various other factors. These factors include species, cultivars, dose and duration, developmental stages, experimental conditions and various environmental variables. In addition, as there are not many data available regarding the interactive effect of heavy metals and UV-B on medicinal plants, it is not completely clear whether the two stresses (UV-B and heavy metals) act synergistically or antagonistically. However, the studies reviewing this interactive effect mostly showed a synergistic effect on the formation of secondary metabolites in medicinal plants. Furthermore, there is a need to explore interactive studies involving different doses of heavy metals and UV-B radiation on various medicinal plants under varied environments to unravel the precise mechanism behind this stimulation. Moreover, understanding how UV-B and heavy metals both individually and in combination influence the accumulation of secondary metabolites in medicinal plants can help us to assess these metabolites’ biological functions, and can also offer standards for specifying the ideal quality and yields of medicinally important compounds. Hence, the future implementation of various omics technologies should significantly advance our fundamental understanding of UV-B and heavy metal-induced responses in medicinal plants. 

## Figures and Tables

**Table 1:** Effect of UV-B on secondary metabolite accumulation in medicinal plants.

Medicinal Plants	Active Metabolites	Medicinal Properties/Uses	UV-B Dose and Duration	Responses	References
*Glycyrrhiza uralensis* L.	Glycyrrhizin	Natural sweetener, antitumor and anti-HIV activity	High intensity UV-B radiation (3 days at 1.13 W m^−2^). Low intensity UV-B radiation (15 days at 0.43 W m^−2^)	Both high and low intensity UV-B treatment increased glycyrrhizin content by 1.5 fold	[26]
*Prunella vulgaris* L.	Rosmarinic acid, caffeic acid and hyperoside	Thyroid gland malfunction, sedative and antifebrile agent	35 μW cm^−2^ nm^−1^ 30 min for 15 days	Caffeic acid, rosmarinic acid and hyperoside increased by 22.5, 53.4 and 121%, respectively	[27]
*Eclipta alba* L. (Hassk)	Wedelolactone	Liver cirrhosis, hepatitis and baldness	Intermittent and continuous exposure to UV-B (ambient + 7.2 kJ m^−2^ d^−1^) for 130 and 240 h, respectively	Wedelolactone content increased by 74.5 and 8.3%, respectively at intermittent and continuous exposure	[28]
*Acorus calamus* L.	β-asarone, aristolene and caryophyllene oxide	Antispasmodic, carminative, anthelminthic, hypotensive and anti-depressant properties	Ambient + 1.8 kJ m^−2^ d^−1^ for 3 h	β-asarone decreased by 8.4% whereas aristolene and caryophyllene oxide increased by 47.6 and 66.6%, respectively	[29]
*Withania somnifera* Dunal	Withanolide A and withaferin	Nervine tonic, antiarthritic, immune modulator and anticancerous activities	Ambient + 3.6 kJ m^−2^ d^−1^ for 3 h	Withanolide A decreased by 41.2% in leaves whereas withaferin A increased by 12.4% in above-ground parts	[30]
*Rhododendron chrysanthum* Pall.	Flavonoids	Inflammation, pain, skin ailments, common cold and gastrointestinal disorders	2.3 W m^−2^ for 8 h	Flavonoids content increased by 62%	[31]
*Glycyrrhiza uralensis* L.	Apigenin, flavonoids	Anti-inflammatory, antiviral, antimicrobial, antioxidative and anticancer	0.024 W m^−2^ for 12, 24, 48 and 96 h	Apigenin content increased by tenfold as compared to control with 12 h irradiation of UV-B	[32]
*Conyza blinii* H. Lev.	Blinin	Anti-inflammatory, analgesic, antitumor	20, 40 and 80 μW cm^−2^	Blinin content increased under all doses of UV-B	[33]
*Perilla frutescens* L	Rosmarinic acid and anthocyanins	Antibacterial, antioxidant	4 W m^−2^ with continuous irradiation (4 W 24 h) and 6 W m^−2^ with 16 h irradiation (6 W 16 h) for 3 d	Rosmarinic acid contents in 4 W 24 h and 6 W 16 h treatments were 103 and 168% higher however, anthocyanin content decreased	[34]
*Acanthopanax senticosus* Rupr. and Maxim	Eleutheroside B and E (saponin)	Anti-diabetic, neuroprotective, anti-inflammatory, cardioprotective	1.9 kJ m^−2^ d^−1^ (medium dose) and 3.4 kJ m^−2^ d^−1^ (high dose) for 2 h	Eleutheroside B and E content increased by 1.5 and 1.9 times under a medium dose and by 1.9 and 3.5 times at a higher dose	[35]
*Vaccinium corymbosum* L. cv. Brigitta and Bluegold	Delphinidin (anthocyanins) and chlorogenic acid (phenolic acid)	Antioxidant	0.07, 0.12 or 0.19 W m^−2^ UV-B radiation for 0, 6, 24, 48 and 74 h.	Delphinidin was abundant in Bluegold and increased by 175% as compared to control at the highest dose whereas chlorogenic acid increased by 83% in Brigitta at a lower dose	[36]
*Artemisia annua* L.	Artemisinin (sesquiterpene lactone)	Antimalarial	2.8 W m^−2^ of UV-B radiation for different short-term (1, 2, 3 and 4 h)	Artemisinin content increased by 103% after 3 h of UV-B irradiation	[37]
*Sinopodophyllum hexandrum* Royle	Podophyllotoxin	Anticancer	107 μW cm^−2^ irradiance at 306 nm;	Podophyllotoxin showed 0.79- and 0.86-fold decreases under UV-B on a dry weight and per plant basis, respectively	[38]
*Astragalus membranaceus* L.	Isoflavonoids	Antioxidant, cardioprotective, anti-inflammatory, antiviral and immunomodulatory	5.4–172.8 kJ m^–2^ d^–1^ for 0.5–16 h	Under optimal elicitation dose (86.4 kJ m^–2^ d^–1^), total isoflavonoids content increased by 2.29-fold against the control	[39]
*Achyranthes bidentata* Blume	Oleanolic acid and ecdysterone	Anti-inflammatory, anti-arthritic, diuretic and antibacterial agent	Duration (dose): 1 h (0.738 kJ m^−2^), 2 h (1.476 kJ m^–2^), 3 h (2.214 kJ m^−2^) and 4 h (2.952 kJ m^−2^)	The greatest increase in oleanolic acid was observed in the 3 h UV-B treatment with an increase of 201.53%, a peak increase of 255.54 and 347.96% total ecdysterone content in leaves and roots, respectively was observed after 2 h of UV-B treatment	[40]
*Panax ginseng* CA Meyer	Ginsenoside	Antidiabetic, anticancer, neuroprotective and immunomodulatory effect	0.36, 0.72 and 1.08 kJ m^−2^ d^−1^	Non- significant variation was observed in ginsenoside content under UV-B	[41]
*Chlorophytum borivillianum* Sant. et. Fer.	Saponin	Aphrodisiac, revitalizer, sex tonic and remedy for diabetes	Low (ambient + 3.2 kJ m^−2^ d^−1^) and high (ambient + 7.2 kJ m^−2^ d^−1^) UV-B dose	Under low dose of UV-B, content of saponin enhanced by 26%	[42]

**Table 2:** Effect of heavy metals on the accumulation of secondary metabolites in medicinal plants.

Plants	Active Metabolites	Importance	Heavy Metal	Effect	References
*Mentha piperita* L.	Menthol, menthone and menthyl acetate	Essential oil is used as an antispasmodic, carminative, analgesic, antimicrobial, antiviral and vasodilating agent	30, 60 and 120 mg kg^−1^ Cd in the soil	All the three levels of Cd enhanced the essential oil concentration; however, Cd-60 increased essential oil concentration maximally by up to 16.1%. Menthol concentration declined to 3.2% by Cd-120 treatment; however, Cd-30 and Cd-60 showed an increase of 8 and 1.03%, respectively. The content of menthone and menthyl acetate increased maximally at Cd-60 and Cd-120, respectively	[68]
*Centella asiatica* L.	Asiaticoside, madecassoside, asiatic acid and madecassic acid collectively known as centellosides	Nootropic disorders, memory loss, insomnia and dermatological issues	50, 100, 150 and 200 mg kg^−1^ Cd in the soil	In terms of total centelloside content, maximum content was recovered from leaves from Cd-200 treatment. However, Cd-50 and Cd-100 treatments failed to elicit the production of centellosides	[62]
*Tanacetum parthenium* L.	Camphor	Essential oil exhibits anti-inflammatory, antioxidant, antifungal and insecticidal effects	5, 35 and 70 μM Cd/Cu	Exposure to Cd at the level of 5, 35 and 70 μM decreased essential oil content by 21, 38 and 41%, respectively. However, essential oil content of plants exposed to 5 μM Cu increased by 6% compared to control plants. The relative content of camphor as the major component decreased by 15 and 12% under 70-μM-Cd and 5-μM-Cu treatments, respectively	[70]
*Panax notoginseng* (Burk.) F.H. Chen.	*Panax notoginseng* saponins (PNS)	Promotes blood circulation, counteracts blood stasis, relieves swelling and pain,	2.5, 5.0 or 10 mM Cd	Cd significantly decreases the accumulation of PNS in the rhizome and main root while promoting the accumulation of PNS in the rootlet	[64]
*Catharanthus roseus* L.	Vincristine, vinblastine, ajmalicine, vindoline and catharanthine	Anticancer	50 and 100 μM Ag	Vindoline, catharanthine, vincristine, vinblastine and ajmalicine contents increased significantly under all employed treatments after seven days of treatment	[12]
*Althaea* *officinalis* L.	Phenolics	Anti-inflammatory, antimicrobial, antitussive, demulcent and immunomodulatory	0.5, 1 and 2 mM Cu	Treatment with 0.5 mM CuCl_2_ led to 1.67 times higher total phenolics	[71]
*Salvia miltiorrhiza* Bunge	Phenolic acid (rosmarinic acid, caffeic acid ferulic acid, salvianolic acid B, danshensu and cinnamic acid) and tanshinones	Cholestatic liver injury, anti-platelet, cardioprotective and anti- inflammatory effects	15 μM Ag	Rosmarinic acid, caffeic acid and ferulic acid were significantly increased, while accumulations of salvianolic acid B, danshensu and cinnamic acid were decreased. Further, the content of tanshinones also increased	[58]
*Artemisia annua* L.	Artemisinin (sesquiterpene lactone)	Antimalarial	1500, 3000 and 4500 μg L^−1^ As	The concentration of artemisinin increased in a dose- and time-dependent manner, the maximum being 22% at 4500 μg L^−1^ as of 7 days over the control	[61]
*Ocimum tenuiflorum* L.	Eugenol	Antiseptic, antispasmodic and antibacterial and insect repellent properties	10.0, 20.0, 50.0 and 100.0 μM Cr	All the chromium concentrations increased eugenol content by14.46, 24.61, 16.80 and 3.83% under 10, 20, 50 and 100 μM Cr, respectively	[72]

## Data Availability

Data is not publicly available due to privacy.
